# The Effect of Neuroticism Level on Restrained Eaters’ Thinness Fantasy and Attentional Bias for Food

**DOI:** 10.3389/fpsyg.2019.01850

**Published:** 2019-08-13

**Authors:** Jihyang Kim, Kiho Kim, Jang-Han Lee

**Affiliations:** ^1^Department of Psychology, Chung-Ang University, Seoul, South Korea; ^2^Department of Psychology, Chung-Ang University, Seoul, South Korea

**Keywords:** restrained eaters, neuroticism, thinness fantasy, attentional bias, body image

## Abstract

The aim of this study was to examine the role of restrained eaters’ neuroticism level in thinness fantasy and attentional bias for food following exposure to thin-ideal images. Eighty-five female participants were classified into four groups on the basis of their dietary restraint (restrained/unrestrained eaters) and neuroticism level (high/low). They completed self-reports (mood, body dissatisfaction level) on a visual analog scale before and after exposure to thin-ideal images, and then their attentional bias for food was measured using eye-movements. Results indicated that after exposure to thin-ideal images, positive affect was more decreased in restrained eaters with high neuroticism compared to other groups and negative affect was increased in all groups except unrestrained eaters with low neuroticism. Also, restrained eaters with high neuroticism showed a heightened vigilance for food. These findings underscore the role of neuroticism in restrained eaters as a moderating factor of thinness fantasy.

## Introduction

Dietary restraint refers to intentional individual efforts to limit food intake for weight loss or weight control (Herman and Polivy, 1975); the behavior increases risk for onset and maintenance disordered eating such as binge eating and bulimic symptoms (Stice, 2001). Thin-ideal images from mass media are one main risk factor for restrained eating (Anschutz et al., 2011) because restrained eaters are preoccupied with their weight and perceived shape. Images portrayed in the mass media depicting extremely thin and attractive bodies influences restrained eaters, and for some, they set a goal of emulating the thin-ideals portrayed.

When restrained eaters are exposed to thin-ideal images from mass media, these images can sometimes lead to “thinness fantasy” (Mills et al., 2002). Thinness fantasy is cognitive processing that restrained eaters perceive the thin-ideal goal as personally attainable; they believe or fantasize that they can also be thinner in order to achieve the ideal body. This makes them feel thinner, and self-enhancement, such as increased positive affect and body-esteem, and temporarily disinhibit their eating (Mills et al., 2002). For example, restrained eaters who viewed commercial advertisements depicting thin models consumed more food than others who viewed plus size model or product-only advertisements (Warren et al., 2005).

Some replication studies of the impact of thinness fantasy in restrained eaters failed to find evidence of the self-enhancement effect; rather, they showed an increase of negative affect and body dissatisfaction (BD) following exposure to thin-ideal images (Boyce et al., 2013). Individual differences such as neuroticism and conscientiousness, as a moderating factor between exposure to thin-ideal images and restrained eaters’ body images may explain the differential of responses to thin-ideal images of restrained eaters and should be investigated (e.g., Roberts and Good, 2010).

A recent study (Roberts and Good, 2010) posited that neuroticism may be the factor that can explain the inconsistency in the media exposure literature, this is because: only neuroticism listed among the Five-Factor traits was associated with the harmful effect of thin-ideal images. Neuroticism refers to individual differences in emotional lability and adjustment (Costa and McCrae, 1992). When individuals exhibit a high level of neuroticism, they are more likely to be emotionally unstable, experiencing myriad negative affects such as anxiety, hostility, depression, self-consciousness, impulsiveness, and vulnerability (Widiger et al., 2002). Also, they tend to be more reactive to potentially threatening stimuli and prone to avoid them (Rusting, 1998). In the previous study (Roberts and Good, 2010), women with a high level of neuroticism showed a greater decrease of body-esteem and body satisfaction than those with low neuroticism after viewing ideal body images. This is because, in general, exposure to images of exceptionally thin attractive women can be perceived as emotionally threatening to women (Bergstrom et al., 2009) and cause upward social comparison leading to negative affect and low body-esteem. That is, these negative effects appear according to ones’ neuroticism level because individuals with high neuroticism are emotionally unstable and experience upward social comparison more frequently than those with low neuroticism (Van der Zee et al., 1999). The results of previous studies, however, do not suggest that women with low levels of neuroticism may experience increased positive affect following exposure to thin-ideal images. Although the relationship between low neuroticism and positive affect in women has not been clarified thus far, given that neuroticism has positively associated to negative affect and BD, it is expected that compared to restrained eaters with high neuroticism, restrained eaters with low neuroticism may experience more self-enhancement effect after viewing thin-ideal images.

Another important effect of neuroticism on restrained eaters is that it plays a role in the pathological development from restrained eating to bulimia (Heaven et al., 2001). The negative affect that restrained eaters experience after viewing thin-ideal images is related to their dieting goal and thus causes extreme restriction of food intake (Boyce et al., 2013), but they usually fail to maintain their control resulting in overeating. Although most previous studies on media images and food intake in restrained eaters have shown that thin-ideal media images are related to restrained eaters’ food intake, the results are still unclear. Some research has showed increase of food intake after exposure to media images (Mills et al., 2002; Anschutz et al., 2011), while other research showed a decrease of food intake (Strahan et al., 2007), or non-significant effect (Boyce et al., 2013). One reason for the inconsistent results may be that in previous research, taste tests were used not considering whether they had the tendency to restrict food intake. Also, using direct measures (such as taste tests) are likely to be confounded by reporting bias such as demand characteristics (Mills et al., 2002) and social desirability (Brignell et al., 2009). For this reason, in this study, attentional bias for food was measured using an eye-tracking system. Attentional bias is useful to determine the potential mechanism underpinning the relationship between exposure to thin-ideal images and food consumption.

Attentional bias occurs when emotional salient stimuli are preferentially processed compared to neutral stimuli; it also reflects the cognitive and motivational processing of emotional information (Cisler and Koster, 2010). Early attentional bias (vigilance) reflects an automatic process and speeded detection to a salient stimulus (e.g., threat) or the incentive value of the stimulus while later attentional bias (maintenance or avoidance) reflects a strategic process (Berridge, 2009; Cisler and Koster, 2010). In order to demonstrate attention bias toward food cues, several studies have used the Stroop test or a visual probe task (Dobson and Dozois, 2004; Rofey et al., 2004). However, the results of attention bias were limited in explaining both the initial and later stages of attention, as the Stroop test and the visual probe task were not sufficient in identifying these stages of the attention process. Because these measures based on reaction time which provide only an indirect assessment of attention allocation and do not investigate for shifting of attention between stimuli (Miller and Fillmore, 2010). Using an eye-tracking system, vigilance bias can be measured with the gaze direction and initial fixation duration, and avoidance bias can be measured with gaze duration. A previous study (Hollitt et al., 2010) that measured attentional bias for food in restrained eaters have demonstrated that restrained eaters showed a heightened vigilance for food cues but no differences in disengagement compared to unrestrained eaters. As for restrained eaters, food cues potentially threaten their dietary goal, if restrained eaters’ neuroticism is higher, they would show stronger vigilance-avoidance pattern of attention for food.

Overall, the aim of the present study was to investigate whether neuroticism influences mood and BD of restrained eaters following exposure to thin-ideal images and to examine the attentional bias for food in them using an eye-tracker.

## Materials and Methods

### Participants and Screening

Four hundred seventy-five female undergraduate students were recruited, and eighty-five students (*M*_age_ = 22.81 ± 1.59, *M*_BMI_ = 20.13 ± 2.04) ultimately participated in this study. Based on the previous research concerning categorizing participants into four groups based on restraint/neuroticism (Kim and Lee, 2016), participants were selected only if they belonged to the upper or lower 25% of the dietary restraint (a score under 39 or over 51) and neuroticism level (a score under 135 or over 149) using Restraint Scale (RS; Herman and Polivy, 1980) and neuroticism items in Revised NEO Personality Inventory (NEO-PI-R; Costa and McCrae, 1992), respectively. They were divided into four groups: restrained eaters with high neuroticism (R-H), restrained eaters with low neuroticism (R-L), unrestrained eaters with high neuroticism (U-H) and unrestrained eaters with low neuroticism (U-L).

### Materials and Apparatus

#### Restraint Scale (RS)

Restraint Scale (Herman and Polivy, 1980) was used for screening participants. RS consists of 15 items measuring dietary restraint behaviors. Participants responded on a 7-point Likert scale ranging from 0 (not at all) to 6 (very much so). The total score ranged from 0 to 90; higher total scores are indicative of dietary restraint. Cronbach’s alpha in the present study was 0.92.

#### Revised NEO Personality Inventory (NEO-PI-R)

The neuroticism subscale of NEO-PI-R (Costa and McCrae, 1992) was also used for screening participants. This scale includes 48 items and measures an individual’s neuroticism level. Each item is rated from 1 (not at all) to 5 (very much so). The total score ranges from 48 to 240 with a higher the score indicating a greater level of neuroticism. Cronbach’s alpha in the present study was 0.93.

#### Visual Analog Scale (VAS)

A VAS (Heinberg and Thompson, 1995) was used to assess changes in mood and BD level. The VAS consisted of 100 mm horizontal line (0: not at all; 100: very much).

#### Thin-Ideal Images

Thirty commercial images depicting a typical ultra-thin female model were collected from fashion magazines. For validation of the thin-ideal images construct, fourteen female undergraduate students, who did not participate in this study, were asked to rate the attractiveness, valence, and arousal of the images on a 7-point Likert scale ranging from 1 (not at all) to 7 (very much so). Based on the results of the students’ ratings, fifteen images were chosen that were rated relatively attractive. Each image was presented for 20 s on a monitor screen. All pictures were 150 mm high × 100 mm width.

#### Free-Viewing Task

A free-viewing task was used to record the participants’ eye movements to food pictures. Each trial started with a central fixation cross for 1000 ms and then a picture pair was presented for 2000 ms, followed by a blank screen for 500 ms. Participants were asked to look at the pictures on the screen as if they were watching television and to focus on the fixation cross between trials. A total of 64 trials, including four practice trials and 60 main trials (20 critical and 40 filler trials), were conducted. During the critical trials, each of the 10 highly palatable food pictures (e.g., chips) was paired with a matched control picture (e.g., crayons) as closely as possible for physical properties such as color and visual complexity, and then pretested for palatability, valence, and arousal. In addition, 20 pairs of non-food pictures were selected for filler trials and two pairs for practice trials. The stimuli were displayed side-by-side in pairs on a 21-inch wide monitor screen (1680 × 1050 pixels); all pictures were approximately 75 mm high x 138 mm width. The locations of food and control stimuli were counterbalanced across trials. The participants’ eye-movements were recorded by the iView XTM Red-IV Eye Tracking System (SensoMotoric Instruments, Berlin, Germany).

### Procedure

At the beginning of the experiment, participants were provided with an informed consent form and asked to report their positive and negative affect and BD level on a VAS. Subsequently, they were exposed to fifteen thin-ideal images for a total of 5 min, rating the attractiveness of each model. They then reported their positive and negative affect and BD level on the VAS again and performed the free-viewing task. Finally, they were debriefed and provided with a $5 gift card.

### Data Analysis

Attentional bias scores from the eye-movement data were used in the analyses based on previous study (Werthmann et al., 2011). One participant’s data was removed from the eye-movement analysis due to data input error in the eye-tracking system. The direction bias score was calculated for each participant by computing the percentage of initial fixations on the food pictures of all trials on which a first fixation was made to either food or control picture. The bias score greater than 50% reflects a higher proportion of first fixations directed to food cues, that is, vigilance toward food pictures. The initial fixation duration bias score was calculated for each participant by subtracting the mean duration of initial fixations on the control pictures from the mean duration of initial fixations on the food pictures. Positive scores reflect vigilance toward food pictures. The gaze duration bias score was calculated for each participant by subtracting the mean gaze duration on the control pictures from the mean gaze duration on the food pictures. Positive scores reflect attentional maintenance (less avoidance) toward food pictures.

A 2 (restraint status: restrained, unrestrained) × 2 (neuroticism: high, low) Analysis of Variance (ANOVA) was used to analyze participants’ characteristics, mood and BD change and the eye-movement data, including gaze direction, initial fixation duration and gaze duration. SPSS 18.0 for Windows was used for the analyses.

## Results

### Group Characteristics

The mean age and BMI were not significantly different among groups (all *ns*); however, RS and NEO-PI-R scores were significantly different. Regarding the restraint status, R-H group and R-L group had higher scores in RS compared to U-H group and U-L group (*F*(1,81) = 420.51, *p* < 0.001, η_p_^2^ = 0.84). Regarding the neuroticism, R-H group and U-H group had higher scores in NEO-PI-R compared to R-L group and U-L group (*F*(1,81) = 373.04, *p* < 0.001, η_p_^2^ = 0.82). These group differences for restraint and neuroticism were expected given that participants were allocated to such groups based on their scores on these measures. Data on these variables are displayed in Table 1.

**TABLE 1:** Mean (*SD*) values for age, BMI, RS, and NEO-PI-R.

	**Restrained eaters**		**Unrestrained eaters**	
	**High N**	**Low N**	**High N**	**Low N**
	**(*n* = 21)**	**(*n* = 22)**	**(*n* = 21)**	**(*n* = 21)**
Age	22.71 (1.65)	23.14 (1.50)	22.29 (1.62)	23.10 (1.55)
BMI	20.70 (1.98)	20.20 (1.52)	19.86 (2.63)	19.78 (1.93)
RS	62.76 (7.27)	63.55 (8.20)	24.29 (9.63)	26.00 (8.90)
NEO-PI-R	167.33 (9.82)	122.45 (9.61)	167.52 (13.27)	122.38 (9.90)

*High N, high neuroticism; Low N, low neuroticism; RS, Restraint Scale; NEO-PI-R, revised NEO personality inventory.*

### Mood and Body Dissatisfaction Changes

Mood and BD level change was calculated for each participant by subtracting the pre-VAS score from the post-VAS score. A positive score reflects the increase of positive affect, negative affect, and BD level following exposure to thin-ideal images. Four participants’ data and six participants’ data were excluded in positive affect and BD, respectively, because their scores were more than 2 standard deviations greater than the mean of their respective group.

Regarding positive affect, there was a significant interaction between restraint status and neuroticism (*F*(1,75) = 4.12, *p* < 0.05, η_p_^2^ = 0.05), and a significant main effect of restraint status (*F*(1,75) = 6.68, *p* < 0.05, η_p_^2^ = 0.08). A simple main effects analysis indicated that the R-H group reported a greater decrease of positive affect when compared to the R-L group (*F*(1,75) = 5.78, *p* < 0.05) and U-H group (*F*(1,75) = 10.78, *p* < 0.01).

Regarding negative affect, there was a significant interaction between restraint status and neuroticism (*F*(1,81) = 4.10, *p* < 0.05, η_p_^2^ = 0.05). A simple main effects analysis revealed significant differences between the U-L group and U-H group (*F*(1,81) = 5.39, *p* < 0.05) and U-L group and R-L group (*F*(1,81) = 5.85, *p* < 0.05). That is, there were no significant differences in negative affect following exposure to thin-ideal images between individuals with high levels of neuroticism and those with low levels of neuroticism in restrained eaters. Individuals with high neuroticism reported significantly more negative affect than those with low neuroticism in unrestrained eaters.

Lastly, regarding BD, there was a significant main effect of neuroticism (*F*(1,77) = 4.51, *p* < 0.05, η_p_^2^ = 0.06): individuals with high levels of neuroticism reported a greater increase of BD level compared to those with low neuroticism.

### Eye-Movement Data

**FIGURE 1:**
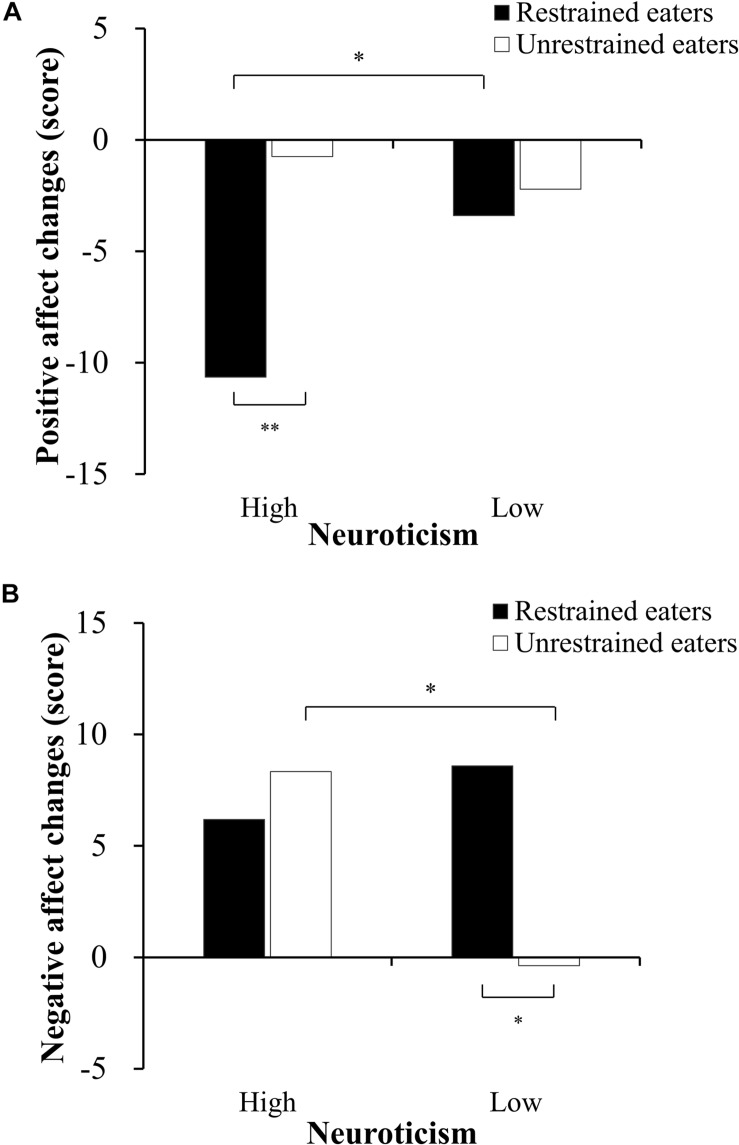
Attentional bias scores for food following exposure to thin-ideal images. Panel **(A)** represents early attentional bias. Panel **(B)** represents later attentional bias. ^*^p < 0.05*, ^∗∗^p* < 0.01.

The gaze direction and an initial fixation duration bias scores were calculated for vigilance bias. There was a significant interaction between restraint status and neuroticism for the gaze direction bias score (*F*(1,80) = 6.37, *p* < 0.05, η_p_^2^ = 0.07). A simple main effects analysis indicated that R-H group and U-L group showed more attentional orienting toward food pictures compared to R-L group (*F*(1,80) = 6.60, *p* < 0.05; *F*(1,80) = 6.34, *p* < 0.05). The results of the gaze direction bias scores are displayed in Figure 1A. There were no significant effects for the initial fixation duration bias score.

The gaze duration bias score was calculated for avoidance bias. There was a significant main effect of restraint status for the gaze duration bias score (*F*(1,80) = 10.51, *p* < 0.01, η_p_^2^ = 0.12). Compared to restrained eaters, unrestrained eaters gazed significantly longer on the food pictures than the control pictures. The results of the gaze duration bias scores are displayed in Figure 1B.

## Discussion

The present study investigated the effect of neuroticism on restrained eaters’ thinness fantasy and attentional bias for food following exposure to thin-ideal images. The changes between pre- and post-VAS scores of mood and BD level revealed that all groups experienced a decrease of positive affect and increase of negative affect and BD, except in the case of unrestrained eaters with low neuroticism in negative affect. In particular, restrained eaters with high neuroticism, when compared to the other groups, showed greatly decreased positive affect following exposure to thin-ideal images, which was consistent with the original study hypothesis. In addition, restrained eaters reported a greater increase of negative affect than unrestrained eaters regardless of their neuroticism level, and individuals with high neuroticism showed a greater increase of BD than individuals with low neuroticism regardless of their restraint status. These results suggest that as one’s dietary restraint is more severe and one’s neuroticism level is higher, the person becomes more likely to experience deleterious effects from thin-ideal images. Thus, the results of the study seem to be consistent with those of recent studies that, suggest: restrained eaters do not exhibit the self-enhancement effect from thin-ideal images (e.g., Boyce et al., 2013) compared with previous research on restrained eaters’ thinness fantasy (e.g., Mills et al., 2002). Especially, this also supports the concept that neuroticism moderates the harmful effect of thin-ideal images in women (Roberts and Good, 2010).

The self-enhancement effect of restrained eaters was not found in the present study. Rather, all restrained eaters showed a decrease of positive affect and increase of negative affect and BD. One possible reason for this is that an individual’s neuroticism, which is susceptible to negative emotions, may not be fully responsible for the increase of positive affect after viewing thin-ideal images in restrained eaters. That is, although neuroticism screening items used in this study measured participants’ level of emotional instability, the individual’s neuroticism level defined as a function of the low score cannot unequivocally represent the person’s emotional stability. Therefore, it is possible that when restrained eaters have emotionally stable trait, such as conscientiousness which is associated with confidence and self-efficacy, (Roberts and Good, 2010), thin-ideal images may increase their positive affect. To investigate this potential relationship, further studies should also consider, in addition to measures of emotional instability, countervailing stable personality traits that can promote positive emotions in restrained eaters.

Based on the analysis of eye-movement data, the R-H group and U-L group directed their initial orientations more often toward food in comparison with the R-L group. In addition, overall attentional maintenance toward food was substantially more intense in unrestrained eaters than in restrained eaters. This attentional pattern observed in the R-H group can be interpreted as heightened vigilance-avoidance for food compared to the gaze pattern of other groups. This suggests that when exposed to highly palatable food, the R-H group may have an initial orientation bias toward food cues but avert their eyes from them to avoid the dietary goal threat (Wegner, 1994). These findings support and extend the results of Hollitt et al. (2010) which examined restrained eaters’ attentional bias for food using a visual search task. In the study, restrained eaters showed heightened vigilance for food cues, but the visual search task used in the study provided indirect evidence of attentional bias, which is only a discontinuous response prior to behavioral responses (Hermans et al., 1999). The present study, however, demonstrated restrained eaters’ early attentional bias and avoidance pattern for food following exposure to thin-ideal images by measuring continuous attentional processing using the eye-tracking system. In particular, the result of early attentional bias for food in restrained eaters suggests that the vigilance bias for food increased along with their neuroticism level.

Overall, the results of the present study indicate that thin-ideal images from media sources can lead to negative mood in restrained eaters leading to vigilance-avoidance pattern for food and BD in individuals with high levels of neuroticism. Thin-ideal images depicting extremely thin and attractive bodies inspire many women, especially restrained eaters, and compel them to set a goal of emulating the thin-ideals that they see on the screen. Generally, this cognitive process induces upward social comparison among the restrained eaters. When women including restrained eaters are exposed to media images of thin-ideal, they compare themselves with the thin-ideal images and focus on areas where they are perceived to be worse. If their goal is higher, it is harder to fulfill, and thus they will have greater self-discrepancy, resulting in increase of negative mood and BD (Krahé and Krause, 2010). According to control theory (Carver and Scheier, 1982) and reinhibition theory (Strauss et al., 1994), the negative mood and BD induced from perceived discrepancy between their present condition and the thin-ideal body triggers goal-related behavior such as restrained eating. In this cognitive process, neuroticism serves as a contributing factor to heightened negative mood and BD because individuals with high levels of neuroticism have more tendencies to make upward comparison than individuals with low levels of neuroticism (Van der Zee et al., 1999; Roberts and Good, 2010). For this reason, it seems that not only R-H group, but also the R-L group and U-H group experienced an increase of negative affect and BD. However, as unrestrained eaters with high neuroticism are not related to dietary restraint, they might not show a vigilance-avoidance pattern of visual attention for food, while restrained eaters with high levels of neuroticism showed the most severe vigilance-avoidance pattern.

Our findings suggest several implications. First, to the best of our knowledge, this is the first study to examine the moderating factor of restrained eaters’ thinness fantasy following exposure to thin-ideal images considering their personality traits. Previous studies have shown mixed results regarding restrained eaters’ thinness fantasy when compared with unrestrained eaters. In the present study, however, participants were divided into four groups according to both their restraint status and level of neuroticism. Although the self-enhancement effect was not observed in the group, restrained eaters with high levels of neuroticism felt less positive affect and more BD than restrained eaters with low levels of neuroticism. These results highlight the importance of neuroticism as a moderator in the relationship between thin-ideal images and restrained eaters. Second, the results of this study may provide indirect evidence of highly neurotic restrained eaters’ goal efforts by identifying a vigilance-avoidance pattern of visual attention for food. Although the gaze duration bias was not significantly different between the R-H group and R-L group, both groups had tendencies to avoid food, and the R-H group showed more intense early vigilance toward food, in line with the results of mood and BD. These results also align with the previous study (Boyce et al., 2013), which suggested that negative affect might encourage restrained eaters’ goal-related behaviors. In future studies, it may be necessary to identify how much either negative affect or BD contributes to highly neurotic restrained eaters’ goal efforts. Finally, the results from the present study could be helpful in clinical settings for designing intervention of patients with excessive restrained eating. Even if restrained eaters experience self-enhancement following exposure to thin-ideal images, in the long term, it can be a maintenance mechanism of restrained eating by seeking out these images to have positive feelings. Thus, it is fundamentally important to change restrained eaters’ ideal body images from unattainable and unrealistic body images to healthy and attractive body images. Additionally, some coping strategies such as emotional regulation or the modification of attentional bias for food should be a consideration for restrained eaters with high neuroticism who are especially susceptible to thin-ideal images. Furthermore, other proper therapies according to weight or shape of restrained eaters or need for health should be considered for future research.

Despite the study’s numerous contributions, there are some notable limitations. First, there were no direct measures of thinness fantasy. Although mood and BD level were utilized as proxies to represent the extent of thinness fantasy based on the previous studies (e.g., Mills et al., 2002; Boyce et al., 2013), future studies should focus on identifying a more direct representation of thinness fantasy such as the Thin Fantasy Scale (Talesfore, 2008). Second, the present study used VAS to measure participants’ mood and BD changes. Although the data showed some significant differences among groups, the self-report data can be distorted due to social desirability or demand characteristics (Mills et al., 2002; Brignell et al., 2009). Given these acknowledged limitations, future studies may benefit from assessing participants’ subjective feelings using indirect methods. Third, this study used a quasi-experimental design rather than an experimental design. A quasi-experimental design is similar to traditional experimental design or randomized controlled trial, but it specifically lacks the factor of random assignment to treatment or control. Thus, in a follow-up study, it would be useful to use an experimental design to compare the effects of different types of images (e.g., thin-ideal versus other body type or neutral images). Finally, this study did not directly investigate the relation between negative affect and attentional bias for food. Although the vigilance-avoidance pattern for food of restrained eaters can be interpreted as their effort to avoid goal threat (Hollitt et al., 2010; Boyce et al., 2013), the results could not conclusively demonstrate the putative relationship. Thus, it would be interesting to investigate how negative affect or BD is related to restrained eaters’ dietary goal and how it influences implicit cognitive processing of food or actual eating behavior.

## Conclusion

In conclusion, the current study demonstrated that restrained eaters with high neuroticism are the most vulnerable to thin-ideal images. They felt negative feelings and showed the most intense vigilance-avoidance pattern for food after exposure to thin-ideal images. These results highlight the importance of clarifying the individual differences moderating the harmful effect of thin-ideal images. Additionally, this study extends our understanding of the role of neuroticism and thinness fantasy in restrained eaters.

## Author’s Note

This study is an extended version of work that was presented in the 17th European Conference on Personality.

## Data Availability

All datasets generated for this study are included in the manuscript and/or the supplementary files.

## Ethics Statement

This study was carried out in accordance with the recommendations of “the Institutional Review Board of Chung-Ang University” with written informed consent from all subjects. All subjects gave written informed consent in accordance with the Declaration of Helsinki. The protocol was approved by the “Institutional Review Board of Chung-Ang University.”

## Author Contributions

All authors designed the study, collected the data, interpreted the results, read and corrected the draft versions of the manuscript, and approved the final version. JK drafted the manuscript with supervision of KK and J-HL.

## Conflict of Interest Statement

The authors declare that the research was conducted in the absence of any commercial or financial relationships that could be construed as a potential conflict of interest.
